# Very Short Mitochondrial DNA Fragments and Heteroplasmy in Human Plasma

**DOI:** 10.1038/srep36097

**Published:** 2016-11-04

**Authors:** Ruoyu Zhang, Kiichi Nakahira, Xiaoxian Guo, Augustine M.K. Choi, Zhenglong Gu

**Affiliations:** 1 Division of Nutritional Sciences, Cornell University, Ithaca, New York 14853, USA; 2 Division of Pulmonary and Critical Care Medicine, Joan and Sanford I. Weill Department of Medicine, Weill Cornell Medicine, New York, NY 10065, USA

## Abstract

Cell free DNA (cfDNA) has received increasing attention and has been studied in a broad range of clinical conditions. However, few studies have focused on mitochondrial DNA (mtDNA) in the cell free form. We optimized DNA isolation and sequencing library preparation protocols to better retain short DNA fragments from plasma, and applied these optimized methods to plasma samples from patients with sepsis. Our methods can retain substantially shorter DNA, resulting in an average of 11.5 fold increase in short DNA fragments yield (DNA <100bp). We report that cf-mtDNA in plasma is highly enriched in short-size cfDNA (30~60 bp). Motivated by this unique size distribution, we size-selected short cfDNA, which further increased the mtDNA recovery rate by an average of 10.4 fold. We then detected mtDNA heteroplasmy in plasma from 3 patients. In one patient who previously received bone marrow transplantation, different minor allele frequencies were observed between plasma and leukocytes at heteroplasmic sites, consistent with mixed-tissue origin for cfDNA. For the other two patients, the heteroplasmy pattern is also different between plasma and leukocyte. Our study shed new lights into the architecture of the cfDNA, and mtDNA heteroplasmy identified in plasma provides new potential for biomarker discovery.

Circulating cell free (cfDNA) has been proposed as a universal diagnostic and monitoring biomarker for many clinical applications, including cancer monitoring, prenatal diagnosis, and transplantation allograft rejection^1^,^2^,^3^. Although a number of the current studies investigating cfDNA have focused on cell free nuclear DNA (nDNA) in plasma, emerging evidence suggests that cell free mtDNA (cf-mtDNA) is also involved in disease progression. For instance, elevated cf-mtDNA concentrations have been observed in various diseases such as breast cancer, stroke, and myocardial infarction^4^,^5^,^6^. Furthermore, clinical reports have shown that the release of mtDNA into plasma is involved in immune responses^7^, and increase with aging^8^, suggesting that cf-mtDNA may serve as a biomarker to monitor disease onset and/or progression.

Although the origin of cf-mtDNA remains unclear, it has been suggested that mtDNA is released from apoptotic cells or necrotic cells^9^,^10^. Interestingly cf-mtDNA levels are not always correlated with cf-nDNA levels in certain pathological conditions such as cancer^11^, implying that cf-mtDNA may provide its unique patho-physiological information distinct from nDNA. It has been well reported that the size distribution of cf nDNA peaks at around 167 bp, suggesting cf nDNA may bind to histones and circulate as intact nucleosomes in blood^12^. Unlike nDNA, mtDNA lacks the protection of histones, making it more vulnerable to degradation^13^, and possibly causing cf-mtDNA fragments to be shorter than cf nDNA. Ellinger *et. al*.^14^, demonstrated that the levels of circulating mtDNA fragments (79 bp and 220 bp) were higher in patients with testicular germ cell cancer, compared to the control subjects. Importantly the diagnostic information (*e.g*., receiver operator characteristic (ROC) curve analysis) of 79 bp mtDNA fragments was relatively higher than that of 220 bp mtDNA fragments^14^, implying the importance of short fragment cf-mtDNA in human diseases. However until now cf-mtDNA has been not fully characterized yet.

Next generation sequencing makes it possible to measure the length of individual plasma DNA fragments at single nucleotide resolution. Using this sequence technique, Lo *et al*.^13^ showed that the size peak of plasma cf-mtDNA is ~140 bp, shorter than that of the predominant nDNA molecules in plasma (~167 bp). However, their strategy for cf-mtDNA size profiling was still technically limited by standard DNA library preparation methods^15^, which contain several purification steps with poor recovery rates for short DNA fragment (<100 bp). In addition to measuring the length distribution of cf-mtDNA, deep sequencing also allows us to assess cf-mtDNA variants, including heteroplasmy. Heteroplasmy is defined as the coexistence of different mtDNA sequences within an individual, and has been reported to be implicated in various human diseases^16^,^17^,^18^. While heteroplasmy patterns have been well studied in different organs or tissues^19^,^20^,^21^, their prevalence in cell free format remain unknown.

In this study, we optimized plasma DNA isolation and library preparation protocols in order to retain short DNA fragments. By our optimized protocol, we recovered substantially shorter DNA fragments than reported in previous studies, yielding a more comprehensive size profile of plasma DNA. Our results indicate that the average length of cf-mtDNA is much shorter than previously reported^13^. We further improved the recovery rate of cf-mtDNA by size selecting short DNA fragments in the sequencing library. Using this strategy in combination with massive parallel sequencing, we investigated heteroplasmy patterns in cf-mtDNA and detected heteroplasmy in 3 individuals’ cf-mtDNA. Interestingly, further investigation indicated that one individual who had bone marrow transplantation (BMT) previously was likely to have both donor and recipient-specific DNA in plasma. We observed very different heteroplasmy patterns between white blood cells (WBC) and plasma in this individual, which may indicate that cf-mtDNA originates from a mixture of different organs. Our results also indicate different heteroplasmy pattern between plasma and white blood cell for the other two patients. The sequencing methods developed in this study and our investigation on plasma heteroplasmy provide an interesting opportunity to further investigate the importance of cf mtDNA fragments and plasma heteroplasmy in patients’ health status in various diseases.

## Materials and Methods

### Clinical sample collection and plasma processing

Blood samples were collected from sepsis patients registered in The Weill Cornell Medicine Registry and Biobank of Critically Ill Patients (WCM BoCI). (WCM BoCI)). WCMC BoCI is an ongoing registry that collects demographic and clinical information, and blood specimens from patients admitted into the medical intensive care unit (MICU). WCM BoCI is approved by Weill Cornell Medicine Institutional Review Board (IRB) and is carried out in “accordance” with IRB protocol #1405015116. All adults (age 18 and older) admitted to the MICU are considered for enrollment. The presence of any of the following excludes a patient from study enrollment: 1) Subjects with mental handicaps. 2) Subjects who are unable to provide consent directly and for whom an appropriate legal representative cannot be found to provide consent. 3) Subjects who have previously indicated that they do not wish to be enrolled in this study, (e.g. during a prior admission to the MICU). 4) Subjects admitted to the MICU purely to facilitate comfort care and weaning of medical intervention at end of life. 5) Subjects who are Jehovah’s witnesses or are otherwise unable or unwilling to receive blood transfusions during hospitalization. 6) Subjects with a hemoglobin level of less than 7 g/dL upon admission to the MICU or subjects with rare blood groups, or other antigens that might require minimization of blood draws. 7) Subjects with active bleeding at the time of MICU admission with hemoglobin levels less than 8 g/dL and subjects suffering from acute myocardial infarction with hemoglobin levels less than 8 g/dL. Written informed consent consistent with the research purposes in this proposal is obtained from all of the subjects prior to study procedures. Sepsis was identified according to the 2001 SCCM/ESICM/ACCP/ATS/SIS International Sepsis Definitions Conference guidelines^22^. Blood samples were drawn and transferred into EDTA-coated blood collection tubes within 24 h of study inclusion as previously described^23^,^24^,^25^ processed within 4 h after venipuncture, and was and stored at −80 °C. 50 μl of plasma was then mixed with 170 μl of sterile PBS, followed by brief vortex. The diluted plasma was centrifuged at 700 g at 4 °C for 5 min, and the supernatant (210 μl) was carefully saved by avoiding touching any pellets and the bottom of the tubes with pipette tips. The obtained supernatant was further centrifuged at 18,000 g at 4 °C for 15 min, and the resulting supernatant (200 μl) was carefully saved. Contamination of cells, cell debris, or pellets into supernatant might lead to a significant change of the results^24^.

### Library preparation for plasma DNA

The obtained plasma samples were processed for DNA isolation using an optimization of a previously published standard method^24^. In the standard method: DNA was extracted by DNeasy Blood and Tissue Kit (No. 69504; Qiagen), and indexed DNA libraries were prepared as described in^15^, using the following steps: i) End repair. ii) Adenine tailing. iii) Adaptor ligation. iv) Library amplification. DNA was purified by 1.5X SPRI beads after each step. For our optimized method: DNA was extracted by QIAamp DSP DNA Blood Mini Kit (No. 61104; Qiagen). DNA libraries were prepared by KAPA Hyper Prep Kits (No. KK8502; Kapa), using the following steps: i) End repair and adenine tailing combined in a single step. ii) Proceeded directly to adaptor ligation. iii) Adaptor ligated DNA was purified by 1.5X SPRI beads and amplified by indexed primers. For size selection to enrich mtDNA fragments in the library, the library was size selected from 150 to 190 bp by Pippin electrophoresis (Sage Sciences Blue Pippen). Libraries were sequenced by the Illumina Hiseq platform with paired-end read lengths of 150 bp.

### Library preparation for white blood cell DNA

DNA from WBC was isolated by QIAamp DSP DNA Blood Mini Kit (No. 61104; Qiagen). MtDNA was amplified as two 9 kb long amplicons (primer sequence: Pair1 Forward: 5′-GATATCATAGCTCAGACCATACC-3′; Reverse: 5′-CCACATCACTCGAGACGTAAAT-3′. Pair2: 5′-CTGCTGGCATCACTATACTACTA-3′; Reverse: 5′-GATGTGTAGGAAGAGGCAGATAAA-3′) and the PCR products were mixed with equimolar ratio. Indexed sequencing libraries were prepared by Nextera XT DNA library preparation kit (Illumina, FC-131–1024). Libraries were sequenced by the Illumina Hiseq and Miseq platform with paired-end reads.

### Analysis workflow for Next-Generation Sequencing data

Raw sequencing reads were trimmed by Trimmomatic^26^ to remove adaptor sequence. Cleaned reads were then mapped to hg19 human reference sequence with rCRS mitochondrial reference genome by bowtie2^27^. PCR duplicates were removed with Picard (http://picard.sourceforge.net.). Read pairs with proper orientation, mapping quality >20, and mismatches less than 5% of trimmed read length were retained for downstream analysis. DNA fragment length was inferred from the coordinates of the nucleotides at the end of each pair of reads.

Nuclear mitochondrial DNA segments (NUMTs) in nuclear genome might be mismapped to mitochondrial genome and counted as mtDNA reads. To minimize the effect of NUMTs, reads mapped to mitochondrial genome were remapped to the hg19 reference genome, and we only retained reads that uniquely mapped to the mitochondrial genome for downstream analysis.

### Heteroplasmy identification for white blood cell and plasma

Sequencing data for each position of mtDNA was extracted by Samtools mpileup^28^, bases were further filtered by sequencing quality (> = 20). Heteroplasmy in WBC was defined with following criteria: i) Sequencing coverage >400. ii) Minor allele frequency > = 1%. iii) Minor allele frequency is no less than 0.6% for both strands, and minor allele frequency at both strands is not significantly different (chi-square test). Heteroplasmy criteria are loosed for plasma DNA due to relative low coverage: i) Sequencing coverage >50. ii) Minor allele counts > = 4. iii) Observed at least once in both strands.

To minimize the false positive heteroplasimes introduced by sequencing error, we applied a maximum likelihood based algorithm to take the sequencing quality at each base in each sequence read into account^20^,^21^. At an interested heteroplasimic site, if there were *l* bases with major alleles and *k* bases with minor alleles, and the probability of sequencing error corresponding to the sequencing quality of each base was ε_j_, the likelihood function of the major allele frequency f can be derived by equation (1):


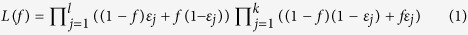


f can be estimated by heteroplasmic model (f_het_) and homoplasmic model (f_homo_) respectively, and log likelihood ratio of these two models can be calculated by equation (2):


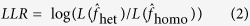


*LLR* >5 indicates a high confidence heteroplasmy (false positive rate <10^−5^). We confirmed that heteroplasmy identified from previous step all had LLR scores >5. Furthermore, heteroplasmy identified from our pipeline were all confirmed by using GATK MuTect2 program^29^.

The strength of a heteroplasmy signal at an mtDNA site may be different between WBC and plasma, due to different mapping criteria. In order to compare heteroplasmy at same sites between WBC and plasma, we defined “heteroplasmy in both WBC and plasma” by the following criteria: i) LLR score >5 in either WBC or plasma. ii) Major and minor alleles need present in both WBC and plasma. iii) Minor allele count > = 2. iv) Minor allele count > = 1 on both strands. Otherwise, the heteroplasmy would be considered as only in WBC or only in plasma.

### Haplotype Analysis

For both WBC and plasma, we constructed two consensus mtDNA sequences, one covering the major alleles at heteroplasmic sites, the other covering minor alleles. We then sent two sequences to HaploGrep^30^ to classify haplogroups. The resulting haplogroups were denoted as major allele haplogroup and minor allele haplogroup respectively.

### Data Access

Sequencing data have been archived in the National Center for Biotechnology Information Gene Expression Omnibus under accession number GSE81178.

## Results

### Plasma mtDNA has a distinct size distribution compared to nDNA

**Figure 1:**
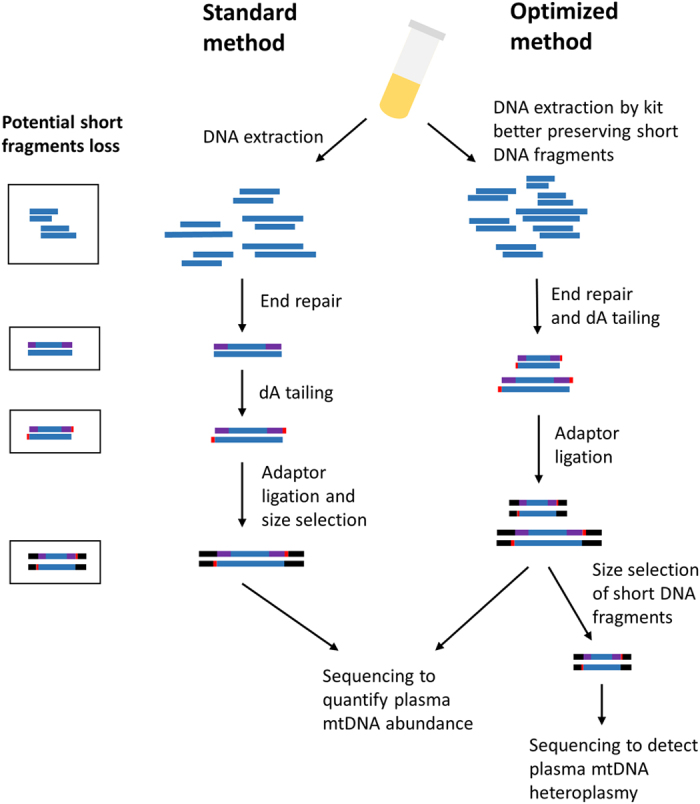
Comparison of the standard method and optimized method. The optimized method has the following improvements: 1. using a DNA isolation kit which can better preserve short DNA fragments 2. combined end repair and dA tailing in a single step, avoiding purification before sequencing adaptor ligation. Black box indicates potential short DNA fragment loss during each step in the standard method. The optimized method produces a 2.41 to 17.88 fold increased in mtDNA concentration. The optimized method can further involve size selection of the ligated library product to enrich mtDNA.

While most of recent plasma DNA extraction methods are column-based, in part due to the need for processing a large number of human samples, short DNA fragment recovery rates are limited. In addition, current standard library preparation protocols include several purification steps with either SPRI beads or columns, which also has poor short DNA fragment recovery rate^31^. Therefore, although these methods are widely used in a range of applications, they are unlikely to capture the complete cfDNA size profile. To circumvent these issues, we optimized these steps in order to better preserve short fragments (Fig. 1): First, we used QIAamp DSP DNA Blood Mini Kit for DNA extraction from plasma, which we verified was able to retain DNA fragments as short as 50 bp (Fig. S1). Second, to avoid any further purification steps before sequencing adaptor ligation, we combined end-repair and dA tailing steps in a single reaction, and then proceeded directly to the adaptor ligation reaction. We also avoided the size selection step which is used in current protocols to remove adapter-dimers, since this may introduce some arbitrary elimination of DNA fragments. To validate the improvement of our optimized methods, we also extract DNA by DNeasy Blood and Tissue Kit and performed standard sequencing library preparation on same plasma sample as a comparison (Fig. 1).

**Figure 2:**
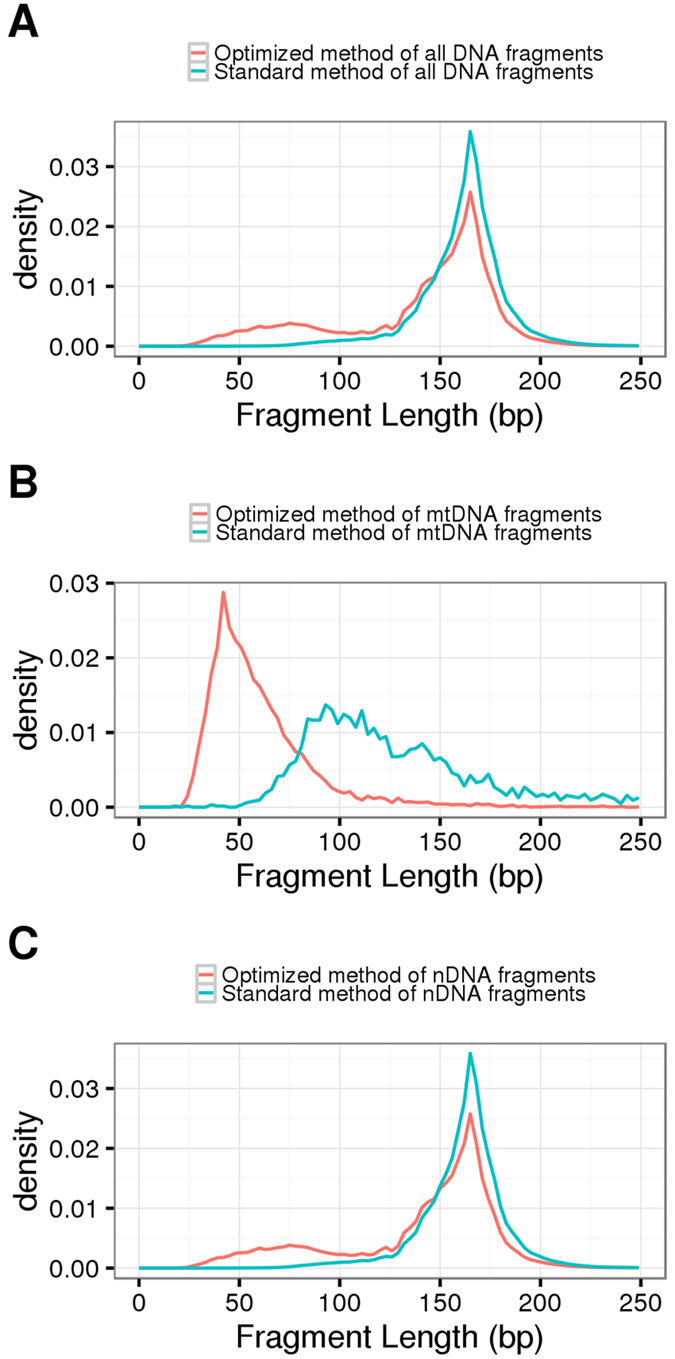
Plasma DNA size distribution. (**A**) The size distribution of all DNA fragments in plasma. Optimized method and standard method were indicated in red line and blue line respectively. Both methods showed a sharp peak at ~ 166–167 bp; in optimized method, 23.2% DNA fragments have length shorter than 100 bp. while in standard method, there was only 1.7% DNA fragments <100 bp. (**B**) The size distribution of plasma mtDNA. The length of mtDNA peaks at 42 bp in our optimized method, and only small fraction of mtDNA had length greater than 100 bp. In standard method, mtDNA length was also shorter than nDNA, but the size peak was ~90 bp, much longer than our optimized method. (**C**) The size distribution of the nuclear DNA in plasma. The size peak was at ~ 166–167 bp, which was consistent with previous reports^12^.

Pair-end sequencing was used to accurately measure the length of each individual DNA fragment. Adaptor sequences were trimmed by Trimmomatic^26^ prior to alignment. Trimmed reads were aligned against the human reference genome hg19, with mitochondrial reference rCRS, using bowtie2^27^. DNA fragment lengths were determined by the paired-end read alignment coordinates. Seven samples were processed by both optimized and standard methods. Compared to the standard method, our optimized method preserved many shorter DNA fragments; the proportion of DNA fragment <100 bp was 19.05% by the optimized method, but only 1.77% by the standard method (Table S1). Figure 2 shows the fragment length distribution of mtDNA and nDNA in both methods. Although by the standard method, cf-mtDNA (Fig. 2b blue line) already shows a left-shifted peak (around 90 bp), our optimized protocol revealed that cf-mtDNA (red line) has a length peak at around 42 bp, much shorter than previously reported^13^. nDNA has a peak at 167 bp (Fig. 2c), consistent with previous studies^12^,^13^. In addition, we noticed that by our optimized method, nDNA fragments also had a flat peak for shorter fragments around 77 bp (Fig. 2c red line), which has not been found in previous studies (Fig. 2c blue line). These results suggest that some nDNA also lacks histone protection, resulting in their degradation and shorter length distribution.

### cf-mtDNA recovery rate is improved by new method

We then compared the mtDNA concentration for the same individual resulting from the optimized and standard methods. We calculated the mtDNA concentration as the ratio of mtDNA reads to the total number of sequenced reads which can be mapped to the human genome (equation (3)).


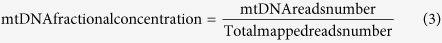


The improvement of the mtDNA recovery rate was shown in Table 1. The fractional concentration of mtDNA in plasma increased by 2.41 to 17.88 fold among different individuals (Table 1). Previously, mtDNA was reported to make up about 0.003% in the plasma cfDNA in hepatocellular carcinoma patients^13^. We obtained a similar average mtDNA concentration of 0.0098% using the standard method. However, in our optimized method, the average mtDNA fractional concentration increased to 0.1428%. These results showed that our optimized method can recover more mtDNA, and thus estimate mtDNA fractional concentration in plasma much more accurately than previous approaches.

### Size selection further improves cell free mitochondrial DNA recover rate

While each cell has two copies of nuclear genome, there are hundreds to thousands of mtDNA copies in a typical human cell. Mutations can be present in all mtDNA copies (homoplasmy) or only exist in a proportion of mtDNA (heteroplasmy). Few studies have investigated mtDNA heteroplasmy in plasma due to the extremely low concentration of plasma mtDNA.

**Figure 3:**
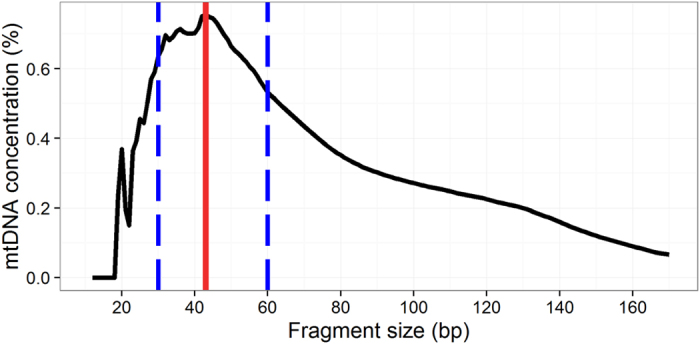
mtDNA fractional concentration in different size windows. In plasma, most of mtDNA exists as short fragments, while the majority of nDNA has larger length. mtDNA thus has relatively high fractional concentration in short size ranges. Blue dashed lines indicate the size region between 30 bp to 60 bp, where mtDNA has highest concentration. Therefore, size selection for plasma DNA from this size region produces substantial enrichment of mtDNA.

Unlike total genomic DNA extracted from cells, plasma DNA is fragmented, therefore it’s difficult to enrich plasma mtDNA by PCR. Since we have discovered that the size distributions for cf-mtDNA and nDNA are different (Fig. 2), we then attempted to use this feature to recover more mtDNA reads. We calculated cf-mtDNA concentration in a series of size intervals by the sliding window strategy. Figure 3 showed that mtDNA fractional concentration varied dramatically in different size intervals, peaking at 43 bp, mtDNA with 0.8%. This number was 10 times higher than the mtDNA concentration across all size range (0.07%). We then size selected DNA molecules (with the ligated sequencing adaptors) between 150 to 190 bp including the ligated sequencing adaptors (corresponding to a DNA insert size of 30 to 60 bp) from the sequencing library in order to increase mtDNA concentration (Fig. 3, blue dash lines).

Our optimized experiment protocols for library preparation improved the mtDNA recovery rate many-fold (Table 1). By size selection, we were able to further increase mtDNA concentration by another 2.99 to 16.27 fold from results without size selection. Compared to the standard method, the overall fold increase is from 17.49 to 128.93 fold for different individuals (Table 1). This improvement of mtDNA recovery rate made it feasible to assess cf-mtDNA heteroplsamy.

### mtDNA heteroplasmy in plasma

Plasma cfDNA can be a mixture of DNA released from different origins (cell types, tissues or organs). In healthy individuals, plasma cfDNA can be derived from hematopoietic cell lineages^9^,^10^, but in disease contexts, multiple organs may be damaged and release cfDNA. Furthermore, since heteroplasmy frequencies can vary from cells to cells and tissues to tissues within the same individual^19^, we suggest that the distribution mtDNA heteroplasmy in plasma can be informative to infer the tissue origins of cfDNA, and from this can inter patients’ disease status.

However, as we indicated above, even with our improved methods, mtDNA only comprises a very small fraction of the total cfDNA in plasma, potentially making it very costly to retrieve enough sequencing reads to estimate heteroplasmy. Nevertheless, by using both our optimized method and size selection, we were able to evaluate heteroplasmy in 3 individuals, (ID: 1, 42, 93, average sequencing coverage 35.9, 503.8, 45.3 respectively). We also sequenced mtDNA in WBC and identified heteroplasmy in these three individuals for comparison (see Methods). Because the sequencing coverage for plasma was lower than WBC, we used less stringent criteria to identify heteroplasmy in plasma (total coverage > = 40, overall minor allele count > = 4, minor allele count > = 1 on each strand).

In patient #1 and 93, we did not identify any heteroplasmy in their WBC using 1% frequency cutoff (see Methods), while we found that patient #1 had heteroplasmy at mtDNA position 11836 and patient 93 had heteroplasmy at position 16111 in their plasma (Table 2). We then manually inspected sequencing details for these heteroplasmy positions in WBC. We found that the minor alleles were indeed presenting but were filtered out because they did not pass the 1% frequency threshold (0.46% and 0.26% respectively). Although the minor allele signals were weaker than those in plasma, we still considered these heteroplasmy were in both sides (see criteria in Methods). We then compared minor allele frequencies between plasma and WBC. The frequencies were 7.1% and 6.9% in plasma, but were lower than 0.5% in WBC (Table 2), indicating heteroplasmy pattern can be different between plasma and WBC.

In patient #42, we identified 12 heteroplasmy in plasma and WBC. Among them, 8 were commonly presented in both WBC and plasma, and 3 were presented in WBC and 1 in plasma (Tables 3, 4, 5). Similar as patient #1 and 93, we also observed different heterolasmy patterns between WBC and plasma. For example, we only observed T (100%) at position 72 in WBC, but we observed high frequency of C alleles in plasma (15.1%). Interestingly, C allele has been shown to be very frequently observed in liver and kidney at this position^19^. Moreover, we noticed among these common 8 heteroplasmy sites, 7 of them had different major alleles between WBC and plasma. For instance, at position 207, major allele was G in plasma (98.2%, with 1.8% A), but in WBC, major allele was A (96.8% with 3.2%G). These differences may indicate that cf-mtDNA is released from multiple tissues and each tissue may contribute to different proportion of cfDNA in plasma.

### The heteroplasmy difference between plasma and WBC could be caused by bone marrow transplantation

Interestingly, the medical record indicated that patient #42 had allogeneic BMT due to hematological malignancy. Thus, plasma cfDNA in this patient could be derived from either the recipient’s’ or donor’s cells/tissues. To analyze the mtDNA haplogroup of this patient, we constructed two consensus sequences, covering either the major and minor alleles at heteroplasmic sites (see Methods). The haplogroup analysis showed that the major allele haplogroups of WBC and plasma DNA were H18 and H2, respectively. One possible explanation for this difference is that WBC and plasma DNA were derived from different subjects (*i.e*., either the BMT recipient or donor). In addition, the minor allele haplogroup of plasma mtDNA was H18, suggesting a proportion of plasma DNA may be released from WBC.

## Discussion

cf-mtDNA has great potential to serve as a biomarker in various clinical situations. A number of studies have used real-time PCR to show that circulating cf-mtDNA is elevated in various disease conditions. However, our results indicate that these methods are not capable of detecting the majority of mtDNA molecules in plasma due to their ultra-short length. In this study, we optimized DNA isolation and library preparation protocols in order to preserve short DNA fragments in plasma. We verified that optimized method was able to capture short DNA fragments (<100 bp), and we found that mtDNA has a very short length in plasma, peaking at 42 bp, which is much shorter than previously reported^13^. Our optimized protocol can increase mtDNA recovery rate by as much as 19 fold. Compared to real-time PCR based methods, our method can quantify mtDNA content in plasma much more accurately.

The endosymbiont hypothesis suggested that the mitochondrion evolved from a bacterial progenitor^32^, therefore mtDNA contains bacterial specific sequence motifs^33^,^34^. Thus, the release of mtDNA into the circulation may cause severe immune consequences, especially when the release is enhanced in specific disease conditions. For example, mtDNA has been shown to increase in fluids in joints of patients with rheumatoid arthritis, and induce inflammation *in vivo*^35^. Furthermore, liver and kidney which may be responsible for eliminating circulating cf DNA are often damaged in critically illness such as sepsis due to systemic inflammation or infection^36^,^37^. Such organ dysfunction can further lead to leveraged DNA releasing. Thus plasma mtDNA would be a better indicator of overall mtDNA status than WBC mtDNA. Nonetheless, few studies have been conducted to evaluate mtDNA heteroplasmy in the cell free form. One technical difficulty is the ultra-low concentration of mtDNA in plasma as well as its unique size distribution. By size selecting short fragments from the DNA sequencing library, we were be able to further enrich mtDNA by up to >100 fold compared to standard methods, enabling us to investigate cf-mtDNA heteroplasmy in three patients.

We observed different heteroplasmy patterns between WBC and plasma in all three patients. Most of the heteroplasmic positions have different allele frequencies between WBC and plasma. In patient #42 who had previously received BMT therapy, 7 out of 12 heteroplasmic positions even had flipped major alleles between WBC and plasma. In general, high doses of chemotherapy and/or radiation are given to patients who plan to receive BMT in order to destroy cancer cells or the defective bone marrow (BM) of the patients. Therefore, after BMT the recipient’s new BM is mostly replaced with the donor’s, implying that DNA in newly generated WBC of the patients (the recipient) is likely to be the same as the donor’s. However, in some cases the recipient’s tumor cells can survive and remain in the BM even after radiation and chemotherapy, which may lead to co-existence of WBC derived from BM of both the recipient and donor. Taken this into consideration, it is not surprising that we observed different haplogroups for WBC and plasma cf-mtDNA. In addition, although the frequencies were relatively low, we could still detect WBC-derived allele in plasma mtDNA, which could be donor-derived mtDNA. mtDNA provides a valuable tool to identify DNA origins, since the high number of nucleotide polymorphisms in mtDNA can allow discrimination between the donor and recipient. cf-mtDNA has not been deeply studied in transplantation medicine yet, but our results suggest that mtDNA has great potential in monitoring allograft health.

Another possible explanation for this inconsistent heteroplasmy between plasma and WBC is that plasma DNA is a mixture of DNA released from different organs or cell types. For example, it has been reported that high levels of heteroplasmy are observed at position 72 in liver and kidney, moderate levels in skeletal muscle, but low levels in all other tissues^19^. In our analysis, the heteroplasmic C allele at position 72 showed a 15.4% frequency in patient #42, and we found that this patient had acute kidney injury when the plasma sample was collected. It is possible that more mtDNA may have been released from the damaged kidneys, contributing to the high level of the C allele in the plasma. This result suggests that patterns of heteroplasmy in plasma can be used to infer cell death in specific tissues or organs, providing more information about patients’ disease status.

Our study characterized important properties of plasma mtDNA, which will inform future plasma DNA studies. Using our optimized experimental protocols, the mtDNA concentration in plasma can be measured more accurately, which can be applied to study changes in plasma mtDNA concentrations in a wide range of diseases such as cancer, stroke and cardiovascular diseases. We also showed that plasma mtDNA can provide information on heteroplasmy that cannot be provided by a single cell type, which can be extended to infer the tissue origins of cfDNA under specific disease conditions, and provide more information about patients’ disease status.

## Additional Information

**How to cite this article**: Zhang, R. *et al*. Very Short Mitochondrial DNA Fragments and Heteroplasmy in Human Plasma. *Sci. Rep.* **6**, 36097; doi: 10.1038/srep36097 (2016).

**Publisher’s note:** Springer Nature remains neutral with regard to jurisdictional claims in published maps and institutional affiliations.

## Supplementary Material

Supplementary Information

## Figures and Tables

**Table 1:** mtDNA fractional concentration by different approaches.

ID	mtDNA concentration (%)			Fold increase in mtDNA yield		
	Standard Method	Optimized Method	Optimized Method with Size Selection	Optimized /Standard	(Optimized with Size Selection) /Optimized	(Optimized with Size Selection) / Standard
1	0.00332	0.02114	0.17842	6.36	8.44	53.73
2	0.00307	0.01428	0.05366	4.65	3.76	17.49
17	0.00543	0.01310	0.15901	2.41	12.14	29.27
42	0.05152	0.92133	2.75686	17.88	2.99	53.51
69	0.00188	0.00688	0.10392	3.66	15.10	55.27
78	0.00114	0.01044	0.14689	9.16	14.07	128.93
93	0.00237	0.01256	0.20437	5.30	16.27	86.29

**Table 2:** mtDNA heteroplasmy in patient 1, 93.

ID	Position	Type	Depth	Allele1	Frequency of Allele1	Allele2	Frequency of Allele2
1	11836	Plasma	56	A	92.9%	G	7.1%
		Cell	3714		99.54%		0.46%
93	16111	Plasma	58	T	93.10%	C	6.90%
		Cell	2663		99.74%		0.26%

**Table 3:** mtDNA heteroplasmy present in both WBC and plasma in patient 42.

Position	Type	Depth	Allele1	Frequency of Allele1	Allele2	Frequency of Allele2
186	Plasma	481	T	2.1%	G	97.9%
	Cell	2325		0.13%		99.87%
207	Plasma	335	A	1.8%	G	98.2%
	Cell	2040		96.8%		3.2%
8425	Plasma	46	A	19.6%	G	80.4%
	Cell	3352		99.9%		0.1%
12127	Plasma	193	A	92.7%	G	7.3%
	Cell	5602		0.4%		99.6%
13708	Plasma	709	A	0.3%	G	99.7%
	Cell	2015		96.7%		3.2%
14364	Plasma	870	A	0.7%	G	99.2%
	Cell	4353		95.6%		4.4%
16126	Plasma	264	T	17.0%	C	83.0%
	Cell	7077		99.8%		0.2%
16129	Plasma	157	A	72.0%	G	27.4%
	Cell	6878		1.2%		98.7%

**Table 4:** mtDNA heteroplasmy present only in WBC in patient 42.

Position	Type	Depth	Allele1	Frequency of Allele1	Allele2	Frequency of Allele2
477	Plasma	452	T	100%	C	0%
	Cell	942		96.1%%		3.9%
3010	Plasma	865	A	0%	G	100%
	Cell	7087		2.2%%		97.8%
14350	Plasma	667	T	0%	C	100%
	Cell	4788		2.5%		97.5%

**Table 5:** mtDNA heteroplasmy present only in plasma in patient 42.

Position	Type	Depth	Allele1	Frequency of Allele1	Allele2	Frequency of Allele2
72	Plasma	1199	T	84.8%	C	15.1%
	Cell	2325		100%		0%
