# Supplementary material for: Very Short Mitochondrial DNA Fragments and Heteroplasmy in Human Plasma
Source: Sci Rep. 2016 Nov 4;6:36097. doi: 10.1038/srep36097 (PMC5095883; doi:10.1038/srep36097)
Supplement: Supplementary Information [file srep36097-s1.pdf]

Very Short Mitochondrial DNA Fragments and Heteroplasmy in Human Plasma

Ruoyu Zhang[1,†], Kiichi Nakahira[2,†], Xiaoxian Guo[1], Augustine M.K. Choi[2,], Zhenglong Gu[1,*]

[1]*Division of Nutritional Sciences, Cornell University, Ithaca, New York 14853, USA*

[2]*Division of Pulmonary and Critical Care Medicine, Joan and Sanford I. Weill Department of Medicine, Weill Cornell Medicine, New York, NY 10065, USA.*

† These authors contributed equally to the work
* To whom correspondence should be addressed: Z.G (zg27@cornell.edu)

Supplementary information:

Subject recruitment:

The Weill Cornell Medicine Registry and Biobank of Critically Ill Patients (WCM BoCI) for the Study of the Biology of Critical Illness is an ongoing registry that collects demographic and clinical information, and blood specimens from patients admitted into the medical intensive care unit (MICU) (IRB #1405015116). All adults (age 18 and older) admitted to the MICU are considered for enrollment. The presence of any of the following excludes a patient from study enrollment:1) Subjects with mental handicaps.
2) Subjects who are unable to provide consent directly and for whom an appropriate legal representative cannot be found to provide consent. 3) Subjects who have previously indicated that they do not wish to be enrolled in this study, (e.g. during a prior admission to the MICU). 4) Subjects admitted to the MICU purely to facilitate comfort care and weaning of medical intervention at end of life. 5) Subjects who are Jehovah's witnesses or are otherwise unable or unwilling to receive blood transfusions during hospitalization. 6) Subjects with a hemoglobin level of less than 7 g/dL upon admission to the MICU or subjects with rare blood groups, or other antigens that might require minimization of blood draws. 7) Patients with active bleeding at the time of MICU admission with hemoglobin levels less than 8 g/dL and subjects suffering from acute myocardial infarction with hemoglobin levels less than 8 g/dL.

Short DNA recovery rate during DNA extraction:

Synthesized oligonucleotides with different sizes (50, 75, 100, 150 bp) were used to verify the short DNA fragment recovery rate for each DNA extraction kit (QIAamp DSP DNA Blood Mini Kit, No. 69504, Qiagen. And DNeasy Blood and Tissue Kit, No. 61104, Qiagen). Using the recovery rate of 150 bp oligonucleotides as reference, QIAamp kit has much better relative performance in recovering short oligonucleotides (50 and 75 bp) comparing to DNeasy kit (Fig S1).

Plasma DNA size distribution:

Although it has been reported that the size peak of plasma DNA is ~166-167 bp (1-3), by our optimized method, we found there are also a proportion of plasma DNA with small fragment length. 19.05% plasma DNA was shorter than 100 bp by our optimized method, but only 1.77% by the standard method (Table S1). These small DNA fragments were overlooked in previous studies.

The plasma DNA size distribution of each individual in this study is shown in Fig S2. mtDNA existed as small fragments (< 50 bp) in each individual.

Table S1 proportion of plasma DNA in different size intervals (%)

Size (bp)	(0,20]	(20,40]	(40,60]	(60,80]	(80,100]	(100,120]
Standard method	0.00	0.01	0.05	0.32	1.39	2.40
Optimized method	0.00	1.56	4.99	6.86	5.64	4.57
Size (bp)	(120,140]	(140,160]	(160,180]	(180,200]	(200,220]	(220,240]
Standard method	7.28	28.85	47.01	8.87	1.89	0.42
Optimized method	10.32	26.91	31.83	4.91	1.02	0.25

Figure S1 Relative recovery rate of DNA fragments with different lengths

Relative recovery rate of DNA fragments with different lengths (recovery rate of 150 bp DNA set as 1). QIAamp has better performance than DNeasy for short DNA fragments (50 bp in this assay)

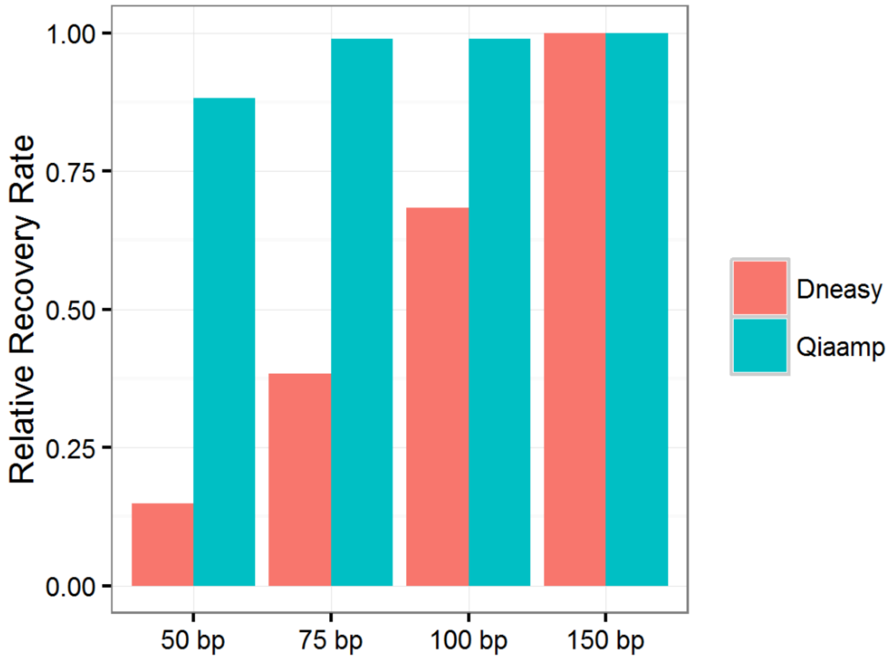

Figure S2 **Plasma DNA size distribution for each individual**
Red line: optimized method, blue line: standard method. (A) Size distribution of all DNA fragments in plasma. (B) Size distribution of mitochondrial DNA. (C) Size distribution of nuclear DNA in plasma.

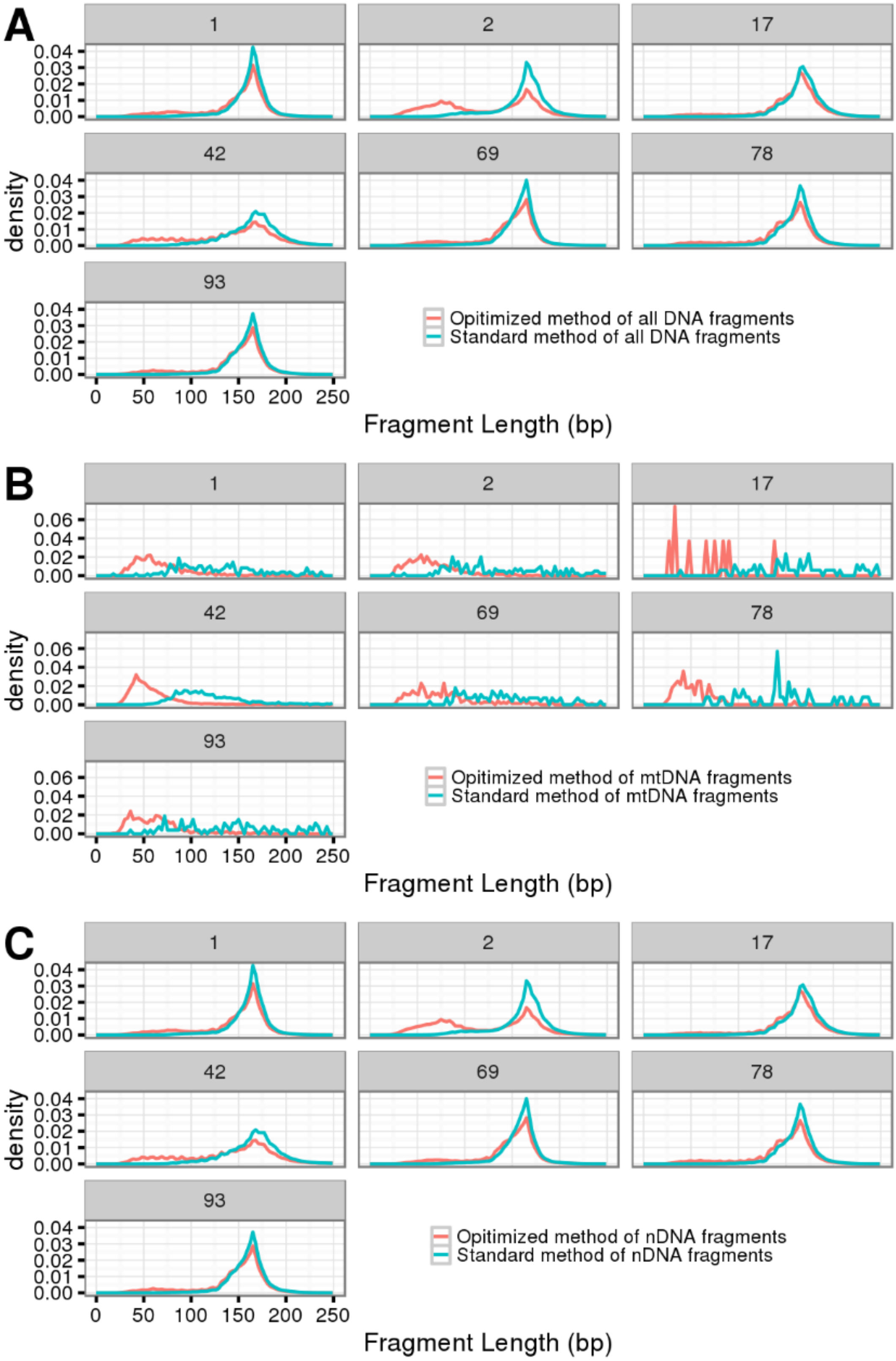

Reference

1. Lo YD, Chan KA, Sun H, Chen EZ, Jiang P, Lun FM, et al. Maternal plasma DNA sequencing reveals the genome-wide genetic and mutational profile of the fetus. Science translational medicine. 2010;2(61):61ra91-61ra91.
2. Jiang P, Chan CW, Chan KC, Cheng SH, Wong J, Wong VW, et al. Lengthening and shortening of plasma DNA in hepatocellular carcinoma patients. Proceedings of the National Academy of Sciences of the United States of America. 2015;112(11):E1317-25.
3. Snyder MW, Kircher M, Hill AJ, Daza RM, Shendure J. Cell-free DNA Comprises an In Vivo Nucleosome Footprint that Informs Its Tissues-Of-Origin. Cell. 2016;164(1-2):57-68.
